# 

**Published:** 1894-12

**Authors:** 


					﻿Vol XIV.
DECEMBER, 1894.
No. 12.
THE
HOMEOPATHIC pHY^lClAfl,
A Monthly Journal of Homoeopathic Materia Medica
and Clinical Medicine.
"He is a freeman whom truth makes free, and all are slaves beside."
“ Seek the Truth: come whence it may, cost what it will."
EDITED AND PUBLISHED BT
WALTER M. JAMES, M. P.
PHILADELPHIA :
3STO- 1231 LOCUST STREET.
iiQ jsrUOTT :
ALFRED HEATH A CO., Sole Agrntu for Great Britain,
114 Ebury Street, Eaton 8quare.
Yearly Subscription, $2.SO, invariably in advance. Single numbers, 2S etc.
Enured at tbe Philadelphia Poat-Offiee u Seooad Clue Mall Matter.
				

